# Path Planning for a Wheel-Foot Hybrid Parallel-Leg Walking Robot

**DOI:** 10.3390/s24072178

**Published:** 2024-03-28

**Authors:** Xinxing Tang, Hongxin Pei, Deyong Zhang

**Affiliations:** College of Mechatronic Engineering, Changchun University of Technology, No. 2055, Chaoyang District, Changchun 130012, China; 2202101106@stu.ccut.edu.cn (H.P.); zdy1979880526@gmail.com (D.Z.)

**Keywords:** walking robot, wheel-foot hybrid parallel-leg, artificial potential field (APF) method, path planning

## Abstract

Mobile robots require the ability to plan collision-free paths. This paper introduces a wheel-foot hybrid parallel-leg walking robot based on the 6-Universal-Prismatic-Universal-Revolute and 3-Prismatic (6UPUR + 3P) parallel mechanism model. To enhance path planning efficiency and obstacle avoidance capabilities, an improved artificial potential field (IAPF) method is proposed. The IAPF functions are designed to address the collision problems and issues with goals being unreachable due to a nearby problem, local minima, and dynamic obstacle avoidance in path planning. Using this IAPF method, we conduct path planning and simulation analysis for the wheel-foot hybrid parallel-legged walking robot described in this paper, and compare it with the classic artificial potential field (APF) method. The results demonstrate that the IAPF method outperforms the classic APF method in handling obstacle-rich environments, effectively addresses collision problems, and the IAPF method helps to obtain goals previously unreachable due to nearby obstacles, local minima, and dynamic planning issues.

## 1. Introduction

Robot construction designs vary significantly based on terrain, with wheeled robots [1], foot robots [2,3], and crawler robots [4,5] representing three commonly used structural types, each with distinct characteristics. Wheeled systems excel in speed and stability on flat surfaces but face challenges on uneven terrain. Foot-type robots, such as those with parallel legs [6,7] or series legs [8,9], offer superior mobility in rugged environments, albeit at the expense of speed and efficiency [10,11]. To address these challenges, Tang et al. [12] introduced a foot-walking robot based on the innovative 6-UPUR + 3P parallel mechanism model, boasting 18 degrees of freedom and enhanced adaptability across diverse terrains. And then, to adapt to various terrains, a wheel-foot hybrid parallel-leg walking robot has been designed to combine the strengths of wheeled and foot robots, enabling independent mode switching.

In the realm of path planning for walking robots [13,14], the artificial potential field (APF) method emerges as a prominent solution due to its simplicity and reliability. Originating from Khatib’s virtual force method [15], APF generates attractive and repulsive potential fields, enabling collision-free navigation [16,17,18]. However, inherent limitations, such as collision issues, unreachable goals near obstacles, local minima, and dynamic environment obstacle avoidance, hinder its effectiveness [19,20,21,22].

To overcome these challenges, innovative solutions have emerged. Pan et al. [23] introduced an improved APF path planning approach incorporating a rotating potential field, effectively circumventing common local minima. Weerakoon et al. [24] and Matoui et al. [25] addressed the local minimum problem using exponential functions and the non-minimum speed algorithm, respectively. Yang et al. [26] proposed a novel repulsive potential function considering the relative distance to the goal, effectively mitigating obstacles near unreachable goals. In dynamic environments, Montiel et al. [27] proposed a parallel evolutionary APF method, while Zhu et al. [28] integrated velocity synthesis and an enhanced APF algorithm to navigate underwater obstacles. Despite these advancements, there is still room for improving obstacle avoidance in the path planning to make it more efficient and straightforward.

Therefore, this paper presents an improved path planning method for a wheel-foot hybrid parallel-leg walking robot by modifying the potential field function, enhancing responsiveness to sensor data, and reducing computational overhead. Key contributions include modifying the attraction potential field function to address collisions, proposing a distance factor to mitigate unreachable goal issues, introducing a valid virtual target point to resolve local minima, and presenting the relative velocity method for dynamic obstacle avoidance. Utilizing an improved artificial potential field (IAPF) approach, the robot autonomously navigates in the obstacle-rich environments, and the IAPF method is validated through simulations and real-world testing. The paper provides a detailed account of the robot’s design, classical APF method principles, modifications, simulation analysis, and experimental validation.

This paper is structured as follows: Section 2 introduces the structure design of the wheel-foot hybrid parallel-leg walking robot. Section 3 describes the principles of the classical APF method. Section 4 presents the modified APF method to address inherent issues in the classical APF method. Section 5 conducts simulation analysis of the modified APF method, comparing it with the classic APF method in scenarios involving static obstacles and moving objects. Additionally, experiments are conducted, and results are analyzed within a controlled environment to support the proposed solution for the inherent issues in the APF method in Section 6. Finally, conclusions are drawn in Section 7.

## 2. Structure Design of Wheel-Foot Hybrid Parallel-Leg Walking Robot

**Figure 1:**
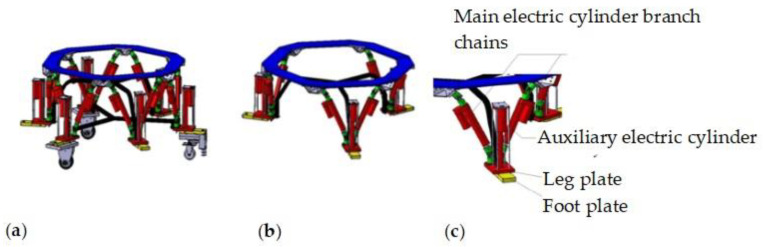
Virtual prototype of wheel-foot hybrid parallel-leg walking robot. (**a**) wheel-foot hybrid parallel-leg walking robot structure. (**b**) Foot-leg structure. (**c**) Single-foot end-branch leg structure.

The structure of the wheel-foot hybrid parallel-leg walking robot comprises two main components: wheel legs and foot legs. The virtual prototype is depicted in Figure 1a.

The foot-leg structure shown in Figure 1b is designed based on a six-degree-of-freedom mechanism, similar to the Stewart platform structure [29,30], and incorporates three auxiliary leg mechanisms. It includes six primary electric cylinder branch chains and three auxiliary electric cylinder branch chains. The upper end of each main electric cylinder branch chain is connected to the upper platform through a universal joint, while the lower end is hinged to the leg-fixing plate via a universal joint seat with bearings. This configuration results in a six-degree-of-freedom structure with a single-chain UPUR distribution. The three leg-fixing plates are integrated through the foot arch frame. Additionally, the auxiliary electric cylinder is firmly connected to the foot’s leg-fixing plate, with the push rod end linked to the foot plate of the auxiliary leg that makes contact with the ground. This design enhances the robot’s Z-direction workspace and its structural adaptability in unstructured environments. A representation of the single-foot end-branch leg structure is presented in Figure 1c.

**Figure 2:**
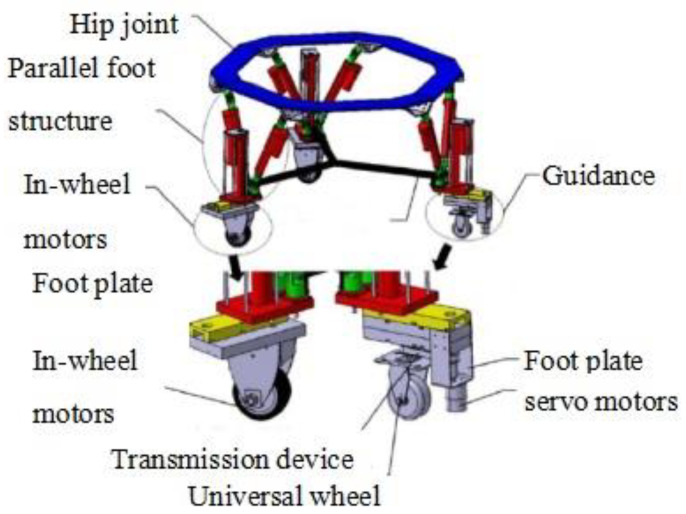
Wheel-leg structure design.

The wheel legs consist of two drive wheels and one guiding wheel, as shown in Figure 2. Their structures are identical to the foot legs, except for the shape of the arch connection frame. Both the wheel and foot legs share the same upper platform, and are referred to as the “hip joint”. These legs are distributed at 180-degree intervals and are perpendicular to the upper platform. The guiding wheel mechanism includes a servo motor, an encoder, a transmission device, and a universal wheel, enabling precise steering control of the universal wheel.

When facing different rugged terrain environments, the wheel control of the wheeled leg locks, and the extension mechanism of the auxiliary leg, can make the wheel of the wheeled leg come into point contact with the ground alternately with the foot of the leg, thereby achieving efficient adaptive walking. For example, when encountering rough and uneven terrain, the foot-leg can utilize its flexibility and stability to assist the wheeled leg in overcoming obstacles. The adjustable extension of the auxiliary leg allows for flexible variations in steps on different terrains, ensuring stable walking of the robot in various complex terrains.

**Figure 3:**
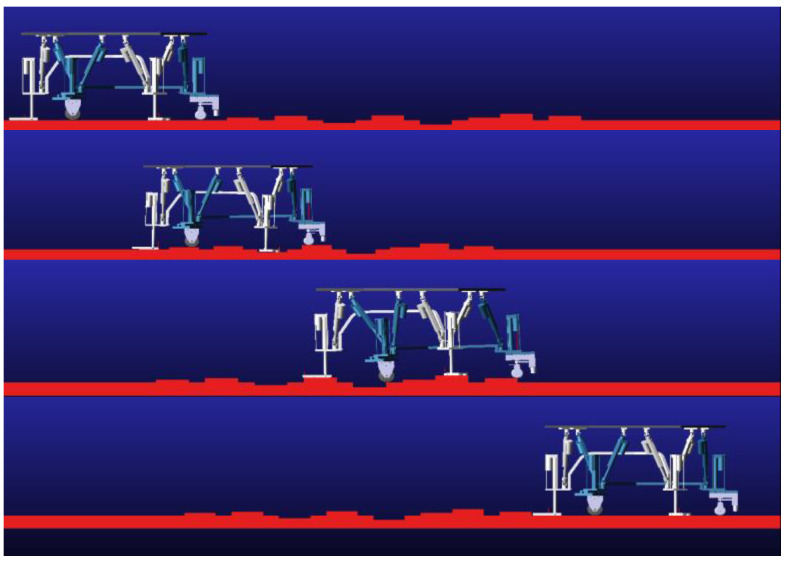
Simulated illustration of the walking performance of a wheel-leg hybrid parallel-leg walking robot in undulating terrain environments.

On the other hand, on smooth walking surfaces, the robot can fully utilize the characteristics of wheeled locomotion to achieve rapid and stable wheeled walking. Through reasonable control strategies, the wheeled leg can quickly adapt to flat ground conditions and advance at a higher speed. Therefore, the synergy between the wheeled leg and the leg enables the robot to flexibly adapt to different terrain environments and achieve efficient walking. Moreover, the extension mechanism of the auxiliary leg, when perceiving small obstacles through sensors such as radar, can directly lift the leg by raising the robot’s body, instead of using avoidance to bypass obstacles. A simulated illustration of the walking performance of a wheel-leg hybrid parallel-leg walking robot in undulating terrain environments is shown in Figure 3.

## 3. Classic APF Principles Method

To address the obstacle avoidance for the walking robot, the APF method is employed. The APF method allows an object to navigate within gravitational and repulsive fields and reach a target point [31,32,33,34,35]. The gravitational potential field function (PFF) *U_att_*(***X***) generated by the target point to robot’s current position can be expressed as:

(1) $$U_{att}(X)=\frac{1}{2}k_{ax}\rho^{2}(X,X_{g})$$

where *k_ax_* represents the positive proportional gain factor of the gravitational PFF *U_att_*(***X***), ***X*** = [*x y*]*^T^* denotes the present position vector of the robot, ***X_g_*** = [*x_g_ y_g_*]*^T^* represents the target point’s position vector, and *ρ*(***X***, ***X****_g_*) signifies the Euclidean distance between the target point position ***X_g_*** and the robot’s present position ***X***.

Based on Equation (1), the gravitational function *Fatt*(*X*) can be derived using the negative gradient potential field method and is given as:

*F_att_*(***X***) = −*∇U_att_*(***X***) = −*k_ax_*(***X***)*ρ*(***X***,***X******_g_***) (2)

In the classic APF method, the repulsion PFF *U_rep_*(***X***) caused by obstacles in the robot’s current position is defined as:

(3) $$U_{rep}(X)=\left\{\begin{array}{ll} \frac{1}{2}k_{rx}{\left(\frac{1}{\rho (X,X_{obs})}-\frac{1}{\rho_{0}}\right)}^{2} & \rho (X,X_{obs})\leq \rho_{0}\\ 0 & \rho (X,X_{obs})>\rho_{0} \end{array}\right.$$

where *k_rx_* is the proportional gain factor of the repulsion PFF *U_rep_*(***X***), ***X_obs_*** = [*x_obs_ y_obs_*]*^T^* represents the obstacle’s position vector, *ρ*_0_ denotes the maximum influence scope of the obstacle, and *ρ*(***X***, ***X_obs_***) represents the Euclidean distance between the robot and the obstacle.

Using Equation (3), the repulsion function *F_rep_*(*X*) can be derivated through the negative gradient potential field method, and is expressed as:

(4) $$F_{rep}(X)=-\nabla U_{rep}(X)=\left\{\begin{array}{ll} k_{rx}\left(\frac{1}{\rho (X,X_{obs})}-\frac{1}{\rho_{0}}\right)\frac{1}{\rho^{2}(X,X_{obs})}\frac{\partial \rho (X,X_{obs})}{\partial X}, & \mathrm{if}\;\rho (X,X_{obs})\leq \rho_{0}\\ 0, & \mathrm{if}\;\rho (X,X_{obs})>\rho_{0} \end{array}\right.$$

where $\frac{\partial \rho (X,X_{obs})}{\partial X}={\left[\frac{\partial \rho (X,X_{obs})}{\partial x}\;\frac{\partial \rho (X,X_{obs})}{\partial y}\right]}^{T}$.

Consequently, in the classic APF environment [36,37], the potential resultant force acting on the robot is given by:

*U*(***X***) = *U_att_*(***X***) + *U_rep_*(***X***) (5)

*F_sum_*(***X***) = −*∇U*(***X***) = *F_att_*(***X***) + *F_rep_*(***X***) (6)

**Figure 4:**
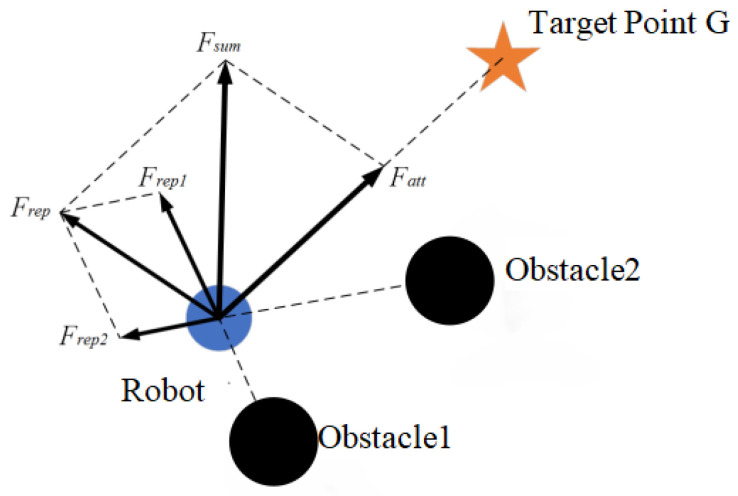
Principle diagrammatic sketch illustrating the APF method.

The repulsion forces generated by Obstacle1 and Obstacle2 on the robot are *F_rep_*_1_ and *F_rep_*_2_ respectively, as depicted in Figure 4. The target point *G* exerts an attractive force on the robot, guiding it toward the point’s direction. As shown in Equation (7), the total resultant force *F_sum_*, which combines the repulsive resultant force *F_rep_* and the attractive resultant force *F_att_*, is the actual force determining the robot’s motion direction for the obstacle avoidance.

*F_sum_*(***X***) = *F_att_*(***X***) + *F_rep_*_1_(***X***) + *F_rep_*_2_(***X***) (7)

When the robot operates in an environment with n obstacles, the potential resultant force acting on the robot should be adapted as:

(8) $$F_{sum}(X)=F_{att}(X)+\sum_{i=1}^{n}F_{repi}(X)$$

where, *F_repi_*(***X***) represents the repulsive force exerted by the *i*th (*i* = 1, 2, …, *n*) obstacle on the robot.

However, the classical APF method has some limitations, including issues with achieving the goal and encountering local minima. Regarding the goal being unreachable, when an obstacle is near the target point, and the target point falls within the influence range of the repulsive potential field generated by the obstacle, the gravitational potential field weakens as the distance between the robot and the target point increases. Simultaneously, the effect of the repulsive potential field intensifies, resulting in a constant repulsive force. Consequently, the resultant force’s direction cannot point toward the target point, trapping the robot within a certain range of the target point and preventing it from reaching it. Concerning local minima, when both the repulsive and gravitational forces on the robot are zero, the combined force is also zero. Even when these forces are non-zero but in equilibrium, the robot can be considered trapped in a local minimum.

## 4. The Proposed Method

### 4.1. Set the Thresholds to Solve the Collision Problem

To tackle potential collisions caused by excessive gravitational force, we enhance the gravitational PFF by introducing a distance threshold, denoted as *d*_0_. When the distance between the robot and the target point exceeds *d*_0_, the attraction force remains at a stable value. However, if the robot’s distance to the target point falls below *d*_0_, the attraction force is calculated using the gravitational PFF from the classic APF. The corresponding attraction PFF is given by:

(9) $$U_{att}(X)=\left\{\begin{array}{ll} \frac{1}{2}k_{ax}\rho^{2}(X,X_{g}) & \rho (X,X_{g})\leq d_{0}\\ d_{0}k_{ax}\rho (X,X_{g})-\frac{1}{2}k_{ax}{d_{0}}^{2} & \rho (X,X_{g})>d_{0} \end{array}\right.$$

Accordingly, the attraction force function is expressed as:

(10) $$F_{att}(X)=-\nabla U_{att}(X)=\left\{\begin{array}{ll} -k_{ax}\rho (X,X_{g}) & \rho (X,X_{g})\leq d_{0}\\ -d_{0}k_{ax} & \rho (X,X_{g})>d_{0} \end{array}\right.$$

### 4.2. Introduce Distance Factor to Solve Goal Unreachable Problem Caused by Obstacles near the Target

The goal unreachable problem arises when the resultant potential field value is non-zero due to the combined effect of the repulsive potential fields from the obstacles and gravitational potential fields from the target point. As the robot approaches the target point, the gravitational potential field gradually weakens. If the repulsive potential field exhibits a similar trend, the robot can smoothly reach the target point. To address this, we introduce a distance factor, which incorporates the real-time distance between the target point and the robot into the classic repulsion PFF to dynamically influence the repulsion potential field. The improved repulsion PFF is given by:

(11) $$U_{rep}(X)=\left\{\begin{array}{ll} \frac{1}{2}k_{rx}\left(\frac{1}{\rho (X,X_{obs})}-\frac{1}{\rho_{0}}\right)\rho^{n}(X,X_{g}) & \rho (X,X_{obs})\leq \rho_{0}\\ 0 & \rho (X,X_{obs})>\rho_{0} \end{array}\right.$$

where *n* is any positive real number.

**Figure 5:**
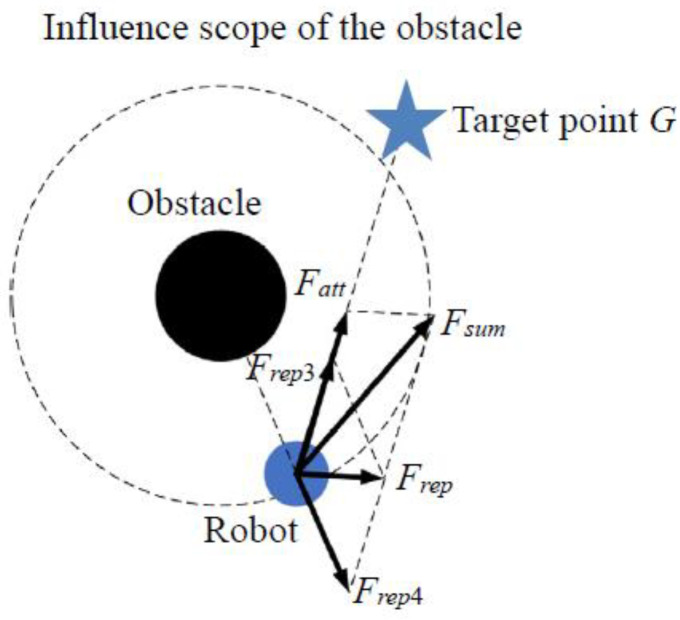
Potential field force diagram of APF method with distance factor.

From Figure 5, it can be observed that the repulsion force exerted by the obstacle on the robot can be broken down into F_rep3_, pointing away from the obstacle, and *F_rep_*_4_, aiming at the target. Consequently, the repulsion function *F_rep_* can be expressed as:

(12) $$F_{rep}(X)=-\nabla U_{rep}(X)=\left\{\begin{array}{ll} F_{rep3}(X)+F_{rep4}(X) & \rho (X,X_{obs})\leq \rho_{0}\\ 0 & \rho (X,X_{obs})>\rho_{0} \end{array}\right.$$

where $F_{rep3}\left(X\right)=k_{rx}\left(\frac{1}{\rho (X,X_{obs})}-\frac{1}{\rho_{0}}\right)\frac{1}{\rho^{2}(X,X_{obs})}\rho^{n}(X,X_{g})\frac{\partial (X,X_{obs})\;}{\partial X}$, $F_{rep4}\left(X\right)=-\frac{n}{2}k_{rx}{\left(\frac{1}{\rho^{2}(X,X_{obs})}-\frac{1}{\rho_{0}}\right)}^{2}\rho^{n-1}(X,X_{g})\frac{\partial (X,X_{g})}{\partial X}$.

Where, $\frac{\partial (X,X_{obs})}{\partial X}$ is the unit direction vector pointing to the robot from the obstacle. This vector indicates that the direction of *F_rep_*_3_(***X***) goes from the robot to the target point; $\frac{\partial (X,X_{g})}{\partial X}$ represents the unit direction vector pointing towards the target point, indicating that the direction of *F_rep_*_4_(***X***) points from the obstacle to the robot.

Due to the influence of the distance factor, as the robot approaches the target point, the repulsion force from nearby obstacles decreases. This reduction effectively addresses the problem of the robot being unable to reach the goal when obstacles are nearby during path planning.

### 4.3. Set Virtual Target Points to Address the Local Minima Issue

In the artificial potential field method, the total force acting on the robot can be expressed as the sum of attraction and repulsion forces, as shown in Equation (6). To find the analytical expression for zero net force, we need to equate the total force to zero. That is

*F_sum_*(***X***) = *F_att_*(***X***) + *F_rep_*(***X***) = 0 (13)

We assumed that the expressions for attraction *F_att_*(*X*) can be represented using a Gaussian function and repulsion forces *F_rep_*(*X*) can be represented using an inverse distance function, as shown in Equations (14) and (15).

(14) $$F_{att}(X)=k_{att}\cdot e^{-\frac{{\left(X-X_{g}\right)}^{2}}{2\sigma^{2}}}\cdot (X_{g}-X)$$

(15) $$F_{rep}(X)=\left\{\begin{array}{ll} k_{rep}\cdot \frac{1}{\rho (X,X_{obs})}\cdot \frac{\left(X-X_{obs}\right)}{\rho (X,X_{obs})} & \left(if\;\rho (X,X_{obs})<d_{0}\right)\\ 0 & (otherwise)\; \end{array}\right.$$

where, *k_att_* is the gain coefficient for attraction, ***X_g_*** is the position of the target point, and σ is the variance of the Gaussian function. *k_rep_* is the gain coefficient for the repulsion, ***X_obs_*** is the current position of the obstacle, *ρ*(***X***, ***X****_obs_*) is the distance between the robot and the obstacle, and d0 is the safe distance.

Combining Equations (13)–(15), then there is

(16) $$K_{att}\cdot e^{-\frac{{\left(X-X_{g}\right)}^{2}}{2\sigma^{2}}}\cdot (X_{g}-X)+k_{rep}\cdot \frac{1}{\rho (X,X_{obs})}\cdot \frac{\left(X-X_{obs}\right)}{\rho (X,X_{obs})}=0$$

This equation represents the balance between attraction and repulsion forces in the artificial potential field method, which will lead to the robot trap in a local mimima point, and result in a failure of the path planning. And Equation (16) can be utilized to determine the position where the robot is in equilibrium. 

When the robot becomes trapped in a local minima point, the resulting force acting on the robot becomes nearly zero, or the robot exhibits small oscillations within a limited range [38]. The conditions for the robot getting stuck in a local minima are as shown in Equation (17):

(17) $$\left\{\begin{array}{l} ||F_{att}(i)-F_{rep}(i)||\leq \Delta f\\ \left|\right|P_{i}-P_{i-2}\left|\right|\leq \Delta p \end{array}\right.$$

where *F_att_*(*i*) and *F_rep_*(*i*) represent the magnitudes of the attraction force and repulsive forces acting on the robot due to the potential field at step *i*, respectively. *P_i_* and *P_i_*_−2_ denote the robot’s location coordinates at step *i* and step *i* − 2, respectively, and *∆f* and *∆p* are small positive real numbers.

If the robot, when assuming a certain position, satisfies any condition in Equation (17), it becomes trapped in a local minima due to a state of balance. Only when this balance is disrupted can the robot escape from the local minima areas. Changing the direction angle of either the repulsive or attraction force acting on the robot results in a change in the direction of the resultant force. This change guides the robot to move in the new direction of the resultant force, allowing it to escape the local minima area. Therefore, a virtual target point is placed near the local minima area. The gravitational potential field originating from this virtual target point disrupts the robot’s balance state in the local minima area and determines the robot’s direction.

**Figure 6:**
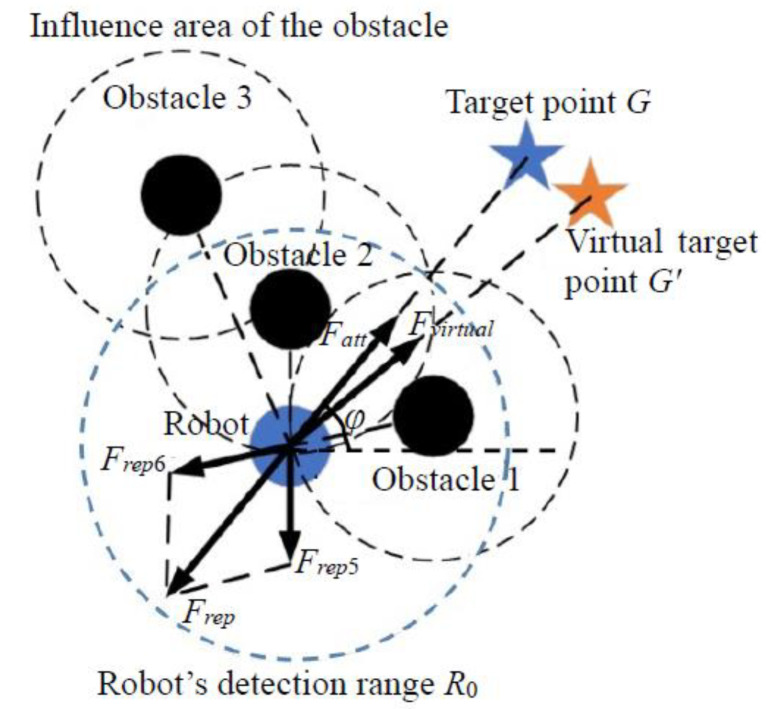
Force diagram of robot after virtual target point generation.

To calculate the angle *φ* between the line connecting the target point and the robot’s current position and the horizontal line, the virtual target point’s location is determined according to Equation (18).

(18) $$\left[\begin{array}{c} P_{virtual\_x}\\ P_{virtual\_y} \end{array}\right]=\left[\begin{array}{c} x\\ y \end{array}\right]+\rho (X,X_{g})\left[\begin{array}{c} \cos(\phi +angle_{add})\\ \sin(\phi +angle_{add}) \end{array}\right]$$

where *P_virtual_x_* and *P_virtual_y_* are the coordinates of the virtual target point, *x* and *y* are the robot’s current position coordinates, *ρ*(***X***, ***X_g_***) is the distance between the robot’s present location and the target point, and *angle_add_* is the angle increment. However, in situations involving multiple obstacles within the robot’s path planning environment, the virtual target point’s position can be determined using the obstacle connection method to overcome the influence of local minima, as illustrated in Figure 6.

Nevertheless, accurately selecting the appropriate *angle_add_* can be challenging, and an inaccurate choice can prevent the robot from escaping the local minima area when using the virtual target point method. Therefore, it may require multiple calculations, significantly increasing the robot’s path planning time and potentially causing the robot to deviate from the target point or collide with obstacles due to multiple virtual target point selections. Moreover, when the robot encounters a complex obstacle environment, such as U-shaped or C-shaped obstacles, traditional methods may fail to plan a viable path. To address these challenges, this paper introduces a symmetrical dynamic virtual target point method, which offers adaptable solutions for various obstacle environments. The specific implementation steps are as follows:

Step 1: Assume that the robot’s detection range forms a circular area with a radius denoted as *R*_0_ (*R*_0_ > *ρ*_0_). When the robot enters a local minimum area, as determined by Equation (13), immediately connect a line between the robot’s current position and the positions of the obstacles within the robot’s detection range. Then, eliminate the positions and count of obstacles exerted within a distance less than *ρ*_0_, as these obstacles exert repulsive forces on the robot.

**Figure 7:**
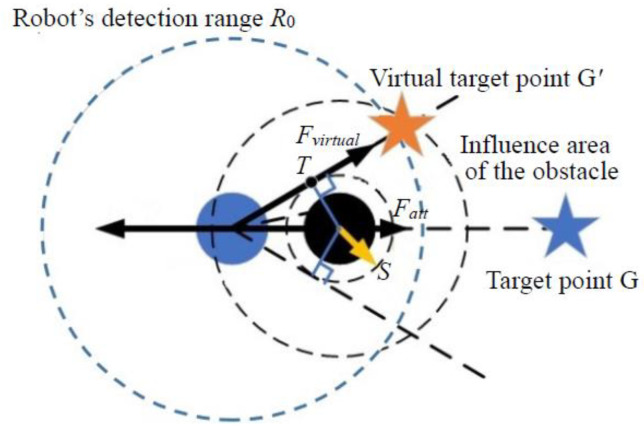
Selection of symmetrical dynamic virtual target point in single obstacle environment.

Step 2: When the number of obstacles within a distance less than *ρ*_0_ is less than 2, meaning that only a single obstacle affects the robot (as shown in Figure 7), the robot, the obstacle and the target point are collinear. Draw a circle centered on the obstacle with a radius denoted as S (S < *ρ*_0_), where S represents the minimum safe collision distance from the obstacle. Draw two tangent lines from the robot’s center to this circle. Since the angle between the two tangents and the line connecting the robot and the current obstacle are equal, either side can be chosen as the direction of movement.

Assuming that the distance between the robot and the tangent point T is Drob_t, the virtual target point’s location is determined by extending the tangent line from tangent point T by a length of Drob_t. The virtual target point generates an attractive force in the direction of the tangent, disrupting the situation where the resultant force is zero. It continuously adjusts as the robot escapes and always maintains symmetry with the robot’s position relative to tangent point T, providing dynamic guidance.

When the robot moves beyond the obstacle’s influence area or no obstacles block the path between the robot and the virtual target point, the original target point is once again selected as the virtual target point.

Step 3: When the number of obstacles within a distance less than *ρ*_0_ is greater than or equal to two, multiple obstacles exert repulsive forces on the robot. In such cases, it can be concluded that the robot is trapped. Without external guidance, the robot will oscillate continuously and remain trapped. Therefore, an obstacle array ***X_obs_*** is established, connecting all obstacles to the robot and sorting them based on the angle between the connection and the *x*-axis. After sorting, the obstacle closest to the robot becomes the *X_om_* benchmark. Obstacles on both sides of the robot are searched and assessed. If the distance to adjacent obstacles is less than 2*ρ*_0_, indicating that the robot cannot pass through the obstacle group, these obstacles are added to ***X_obs_***. The search continues until the distance from the first member *X_os_* or the last member *X_ow_* in *X_obs_* is greater than 2*ρ*_0_, at which point the search stops on that side. At this stage, the obstacles in the array ***X_obs_*** are those that create the minimum trap for the robot. Subsequently, the members in ***X_obs_*** are constantly updated. Whenever the robot enters the influence range of a new obstacle, it determines whether to add it to the end of ***X_obs_***_,_ based on the aforementioned rules. The original target point is then obscured, and the virtual target point is set. The method mirrors that of Step 2, creating two tangents from the robot to the circle with a minimum safe anti-collision distance between the robot and *X_ow_*, and selecting the tangent point *T* direction to ensure it is free of obstacles. The symmetry position *X_xg_* of the robot in the *T* direction, which is the position of the virtual target point, is determined. *X_xg_* also adjusts dynamically with the robot’s motion, and its attractive force guides the robot out of the local minima trap until the robot leaves its influence area or no obstacles obstruct the path between the robot and the virtual target point. At this point, the initially set origin of the path planning becomes the virtual target point once again.

**Figure 8:**
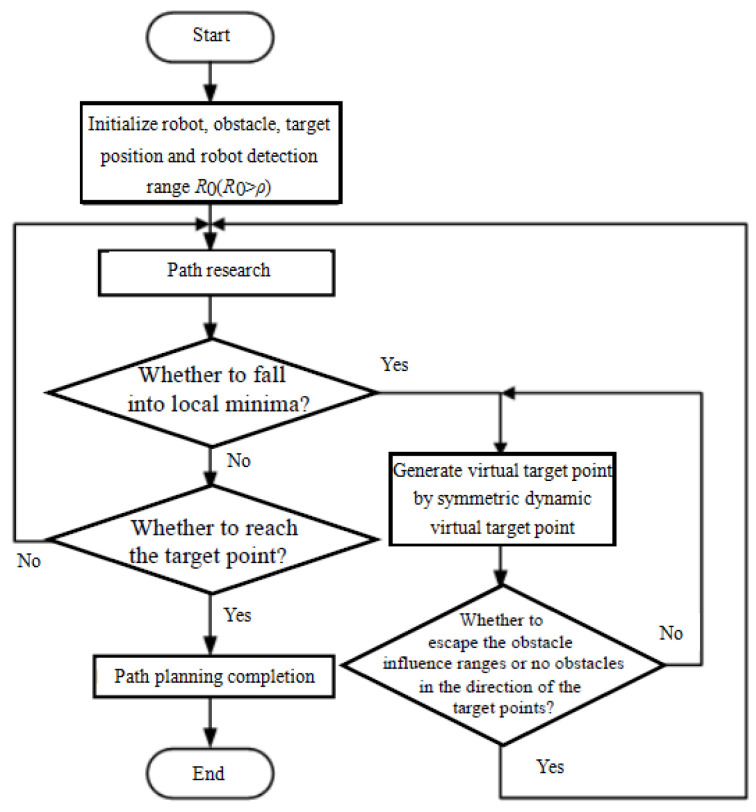
Flowchart of symmetric dynamic virtual target point method for escaping local minima area.

The flowchart illustrating the symmetric dynamic virtual target method for escaping local minima is shown in Figure 8. By introducing the symmetrical dynamic virtual target method to the IAPF approach, the robot can select the virtual target position with greater accuracy. It can also choose virtual target points in different modes, depending on the obstacle environment, greatly enhancing the algorithm’s flexibility.

### 4.4. Introduce Velocity Factor and Acceleration Factor

To enhance the robot’s real-time tracking of dynamic target points, it is imperative to consider the relative velocity and relative acceleration between the robot and the dynamic target point [39,40]. Consequently, the gravitational PFF is refined in the presence of dynamic target points:

(19) $$U_{att}(X,V,a)=U_{att}(X)+U_{att}(V)+U_{att}(a)$$

(20) $$U_{att}(V)=\frac{1}{2}k_{av}|V-V_{g}{|}^{2}$$

(21) $$U_{att}(a)=\frac{1}{2}k_{aa}|a-a_{g}{|}^{2}$$

where *k_av_* and *k_aa_* denote positive proportional gain factors of the gravitational potential field between the robot and the relative velocity and relative acceleration of the dynamic target point, respectively, ***V_g_*** = [*v*_gx_ *v_gy_*]^T^ and ***a_g_*** = [*a_gx_ a_gy_*]^T^ represent the dynamic target point’s velocity and acceleration vectors, respectively, ***V*** = [*v_x_ v_y_*]^T^ and ***a*** = [*a_x_ a_y_*]^T^ represent the robot’s velocity and acceleration vectors, respectively.

Combining Equations (9) and (10) with Equations (19)–(21), the improved gravitational function is expressed as follows:

(22) $$F_{att}(X,V,a)=-\nabla U_{att}(X,V,a)=\left\{\begin{array}{l} -k_{ax}|X-X_{g}|+\frac{1}{2}k_{av}|V-V_{g}|+\frac{1}{2}k_{aa}|a-a_{g}|,\\ \;\;\;\;\;\;\;|X-X_{g}|\leq d_{0}\;\mathrm{and}\;|V_{g}|\neq 0\\ -k_{ax}|X-X_{g}|,\;\;|X-X_{g}|\leq d_{0}\;\mathrm{and}\;|V_{g}|=0\\ -d_{0}k_{ax}+\frac{1}{2}k_{av}|V-V_{g}|+\frac{1}{2}k_{aa}|a-a_{g}|,\\ \;\;\;\;\;\;\;|X-X_{g}|>d_{0}\;\mathrm{and}\;|V_{g}|\neq 0\\ -d_{0}k_{ax},\;\;\;\;|X-X_{g}|>d_{0}\;\mathrm{and}\;|V_{g}|=0 \end{array}\right.$$

where |***X*** − ***X****_g_|* = *ρ*(***X***, ***X_g_***).

Furthermore, enhancements are made to the repulsion PFF, leading to the following formulations:

(23) $$U_{rep}(X,V,a)=U_{rep}(X)+U_{rep}(V)+U_{rep}(a)$$

(24) $$U_{rep}(V)=\frac{1}{2}k_{rv}|V-V_{obs}|$$

(25) $$U_{rep}(a)=\frac{1}{2}k_{ra}|a-a_{obs}|$$

where *k_rv_* and *k_ra_* represent the positive proportional gain factors of the repulsion potential field corresponding to the relative velocity and relative acceleration between the robot and the dynamic obstacle, respectively. Notably, ***V_obs_*** = [*v_obsx_ v_obsy_*]^T^ and *a_obs_* = [*a_obsx_ a_obsy_*]^T^ denote the velocity and acceleration vectors of the dynamic obstacle.

By combining Equations (11) and (12) with Equations (23)–(25), the improved repulsion function can be derived:

(26) $$F_{rep}(X,V,a)=-\nabla U_{rep}(X,V,a)=\left\{\begin{array}{c} \begin{array}{l} F_{rep3}(X)+F_{rep4}(X)+\frac{1}{2}k_{rv}E_{ro}+\frac{1}{2}k_{ra}E_{ro},\\ \;\;\;\;\;\;|X-X_{obs}|\leq \rho_{0}\;\mathrm{and}|V_{obs}|\neq 0\\ F_{rep3}(X)+F_{rep4}(X),\\ \;\;\;\;\;\;|X-X_{obs}|\leq \rho_{0}\;\mathrm{and}|V_{obs}|=0 \end{array}\\ 0,\;\;\;\;\;|X-X_{obs}|\leq \rho_{0}\;\;\;\;\; \end{array}\right.$$

where, |***X*** − ***X_ob_***_s_| = *ρ*(***X***, ***X_obs_***), and ***E_ro_*** represent the unit direction vector pointing from the robot to the dynamic obstacle.

## 5. Simulation Analysis of the IAPF Algorithm

To evaluate the effectiveness of the IAPF method presented in this paper, we establish a local path planning environment in MATLAB 2020a for comparison with the classic APF method. Finally, we perform a quantitative analysis of simulation data. The key parameters used in the simulation are primarily derived from test and simulation environment considerations, with the main parameter settings being detailed in Table 1.

According to the parameters in Table 1, the simulation results for collision, goal unreachability, local minima, and dynamic planning are compared and analyzed, using both the classic APF method and the proposed IAPF method.

### 5.1. Simulation of Collision Problems

**Figure 9:**
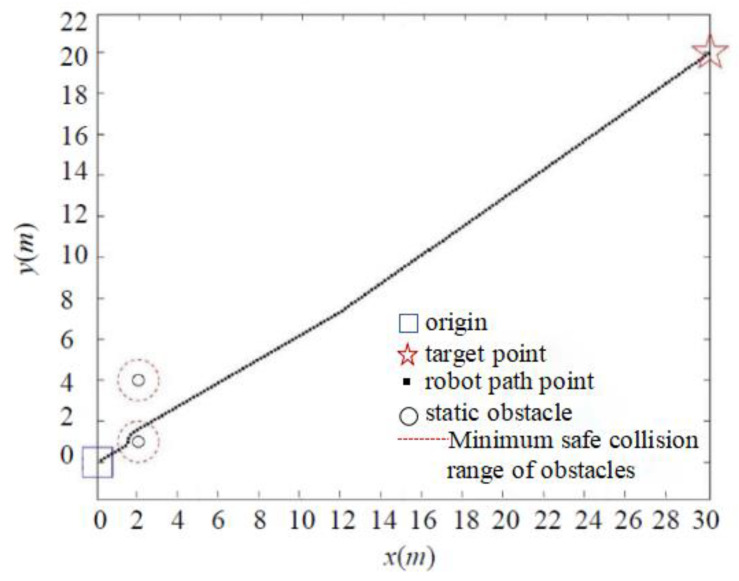
Simulation of classic APF collision problem.

In addressing the collision problem, we present the distance threshold, *d*_0_, which effectively reduces the likelihood of collision resulting from excessive gravitational forces generated by obstacles when the robot is distant from the target point. The simulation results are depicted in Figure 8 and Figure 9.

**Figure 10:**
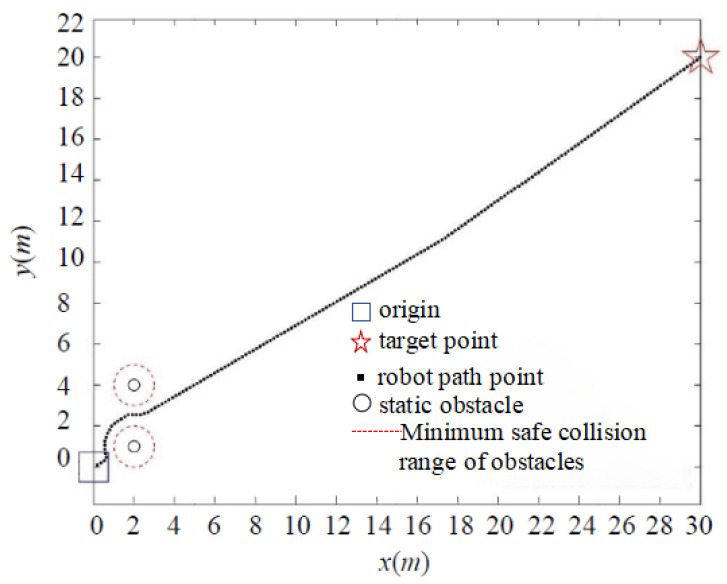
Simulation of collision problem with IAPF method.

As shown in Figure 9, when the target point is far away, the classic APF algorithm enters the minimum safe collision range of the obstacle when encountering a nearby obstacle, due to the significant gravitational pull. Although the target point is eventually reached in the simulation environment, it collides with the obstacle in the real environment. In contrast, due to the presence of the distance threshold, the IAPF method circumvents the minimum collision range of obstacles, as demonstrated in Figure 10, effectively preventing collisions.

### 5.2. Simulation of Goal Unreachable Problem

**Figure 11:**
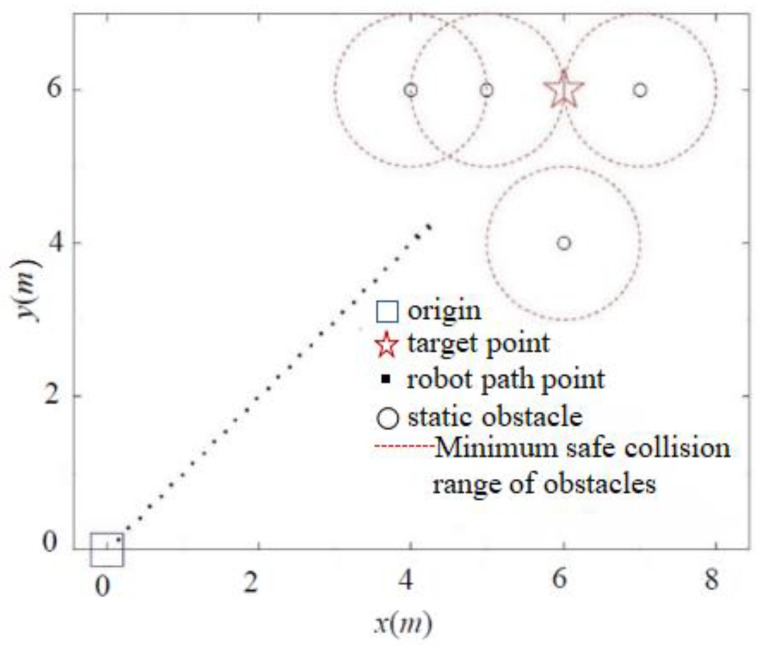
Simulation of unreachable goals problem with the classic APF method.

**Figure 12:**
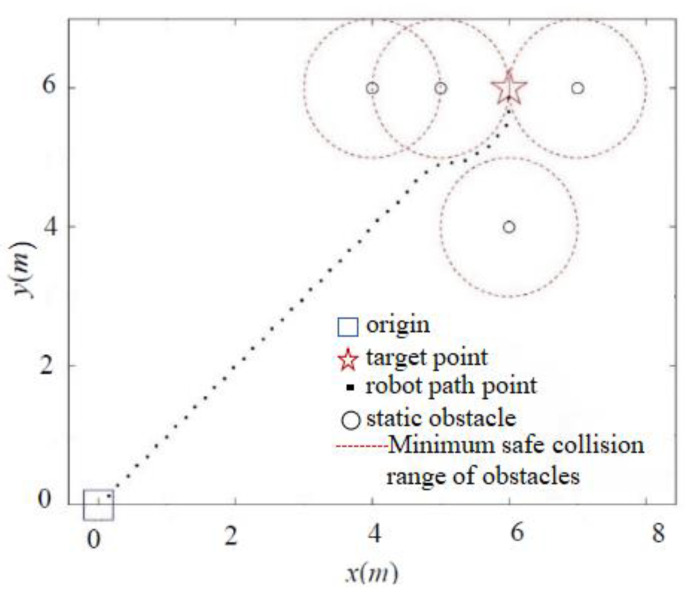
Simulation of an unreachable problem with the IAPF method.

To address the goal unreachability problem, we introduce a distance factor to dynamically adjust the magnitude of the repulsion force, which is based on the distance between the robot and the target point. The simulation results for the goal unreachability problem obtained using the APF and IAPF algorithms are illustrated in Figure 11 and Figure 12.

As depicted in Figure 11, the classic APF method is ineffective in environments where multiple obstacles are close to the target point. In such cases, the influence of gravity is significantly less than the repulsion generated by multiple obstacles, rendering the robot unable to approach the obstacles. In contrast, Figure 12 demonstrates that the IAPF method can dynamically adjust the repulsion force’s magnitude based on the robot’s distance from the target point, ultimately enabling the robot to reach its goal and thereby resolving the goal unreachability issue.

### 5.3. Simulation of Local Minima Problem

**Figure 13:**
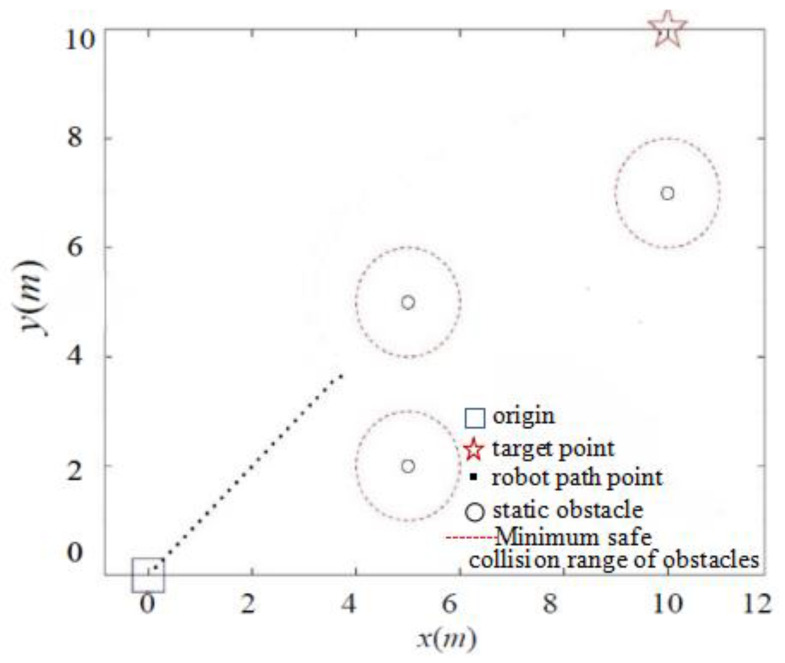
Three-point collinear problem of the classic APF method.

**Figure 14:**
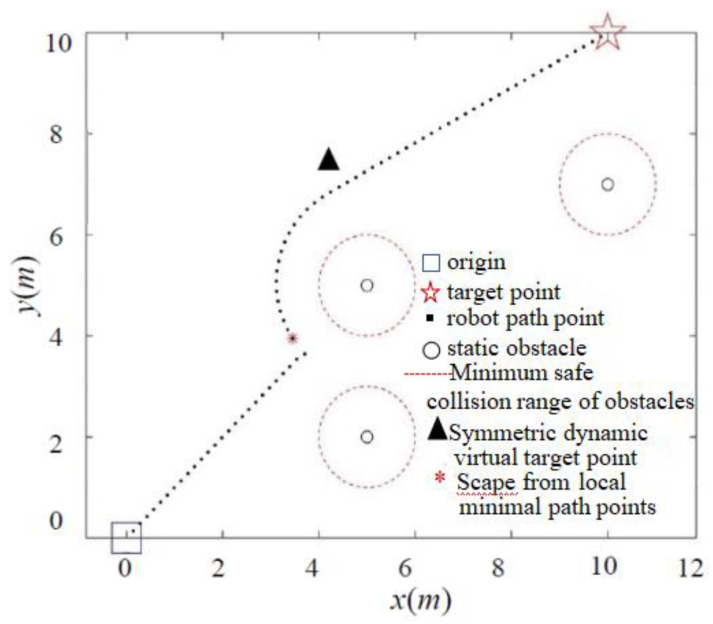
IAPF method to escape from three-point collinearity.

The simulation results for the local minima problem are presented in Figure 13 and Figure 14. In scenarios where the obstacle, target point, and robot are aligned, resulting in a resultant force of 0 on the robot, it becomes trapped in a local minima, as shown in Figure 13. Conversely, as demonstrated in Figure 14, the IAPF method overcomes this issue by generating virtual target points through a symmetrical and dynamic virtual goal method, effectively addressing the problem of three-point collinearity and escaping local minima points.

**Figure 15:**
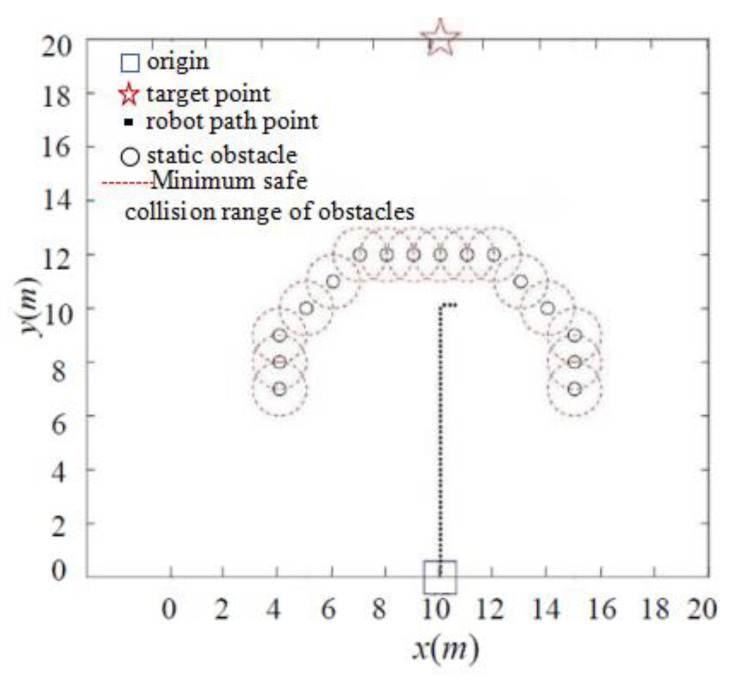
Classic APF method falls into U-shaped obstacle trap.

**Figure 16:**
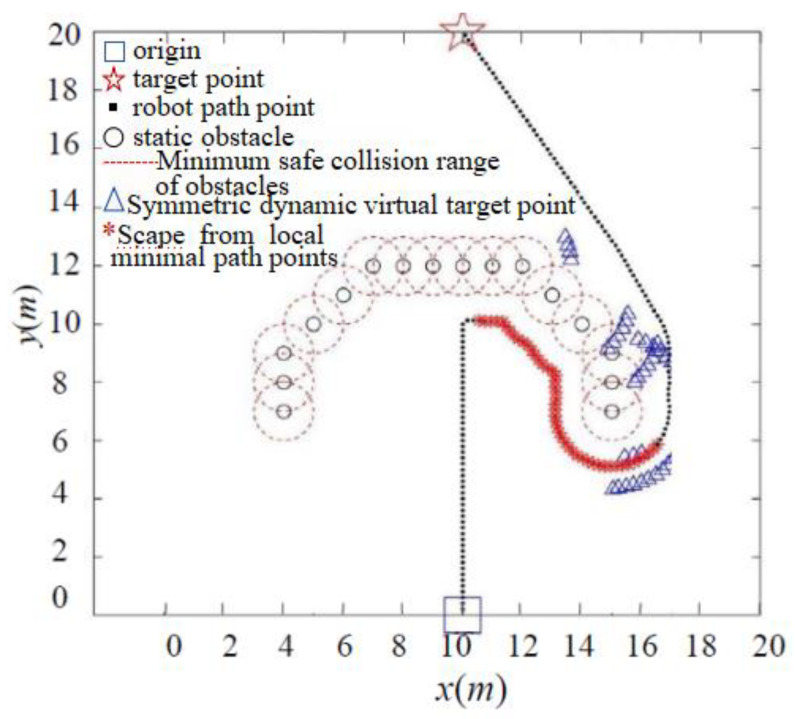
IAPF method to escape U-shaped obstacle trap.

Complex obstacle environments, such as U-shaped obstacles, often consist of multiple connected obstacles that form an impassable trap area for robots, making it challenging to escape. As illustrated in Figure 15, under the classic APF method, the robot becomes ensnared within a U-shaped obstacle area. Because the repulsion and gravity from multiple obstacles continually balance each other, the robot experiences oscillations and remains trapped. In contrast, Figure 16 demonstrates that, by introducing the symmetrical dynamic virtual goal method, the IAPF method shields the role of the original target point when the robot is trapped in a local minima area. The dynamically changing symmetrical virtual target point generates gravitational forces that guide the robot out of the local minima, enabling it to reach the target point smoothly. The method inherits the advantages of the artificial potential field approach, overcomes the oscillation problem, and reduces the probability of falling into local minima. Simulations of robot motion planning were conducted using MATLAB under different gravitational and repulsive forces, demonstrating the effectiveness of the proposed approach.

### 5.4. Simulation of Dynamic Planning

**Figure 17:**
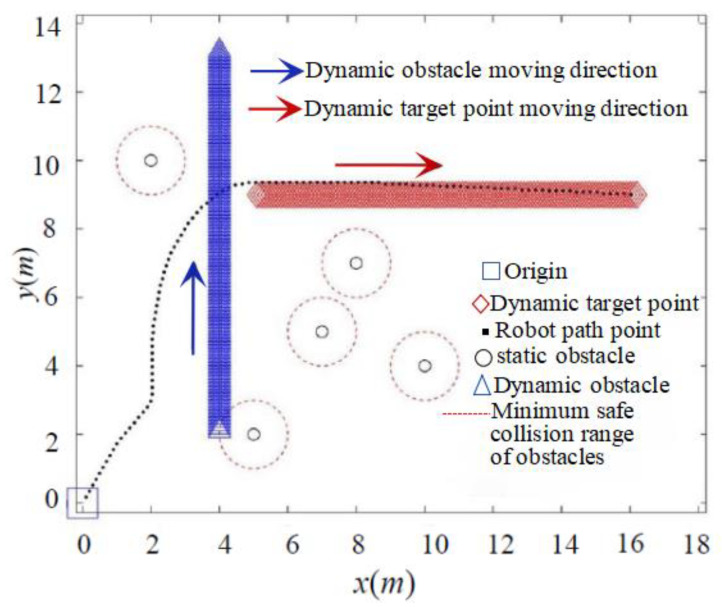
Classical APF method to pursue dynamic goal and avoid dynamic obstacles.

**Figure 18:**
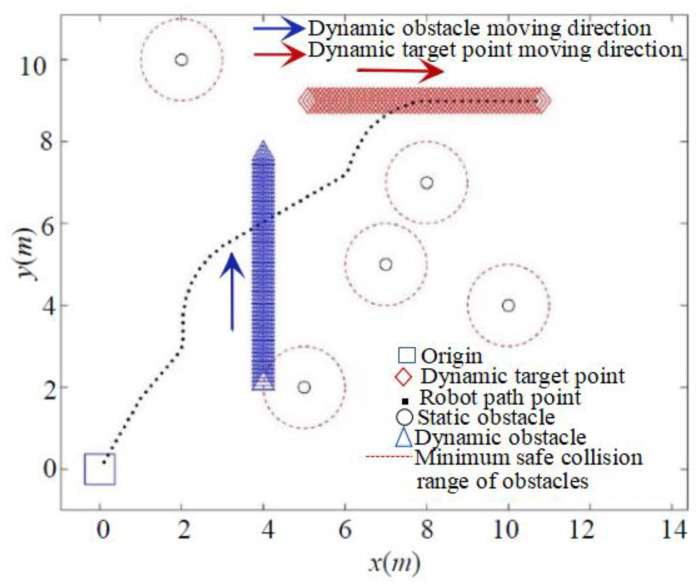
IAPF method to pursue dynamic targets and avoid dynamic obstacles.

As depicted in Figure 17 and Figure 18, both the classic APF and the IAPF method prove effective in pursuing dynamic target points and avoiding dynamic obstacles. However, the IAPF method, by introducing speed and acceleration factors, offers greater flexibility in dynamically avoiding obstacles.

A comparison of the data for both algorithms is provided in Table 2, revealing that the IAPF method enables the robot to pursue the dynamic target point more quickly and with a shorter path. Table 2 presents quantitative data indicators (including time, path length, reduction rates of path length, and running time) for analyzing path planning using the two proposed algorithms. Compared to the classical APF, the IAPF method reduces the path length by 31.25% and the running time by 29.22%, demonstrating improved path search efficiency when dealing with dynamic environments.

The simulation results suggest that the improved artificial potential field method exhibits advantages (such as heightened accuracy, flexibility, stability, and energy efficiency) when addressing collision, unreachable target, local minima, and dynamic planning issues.

(1) Enhanced Path Precision. By introducing distance factors and virtual target points, the method accurately depicts the spatial relationship between the robot and obstacles, refining path planning and bolstering its accuracy.

(2) Reduced Local Optima Risks. Setting distance thresholds for the robot and target point, along with incorporating virtual target points, velocity factors, and acceleration factors, enhances the robot’s obstacle avoidance capabilities. This enables more effective obstacle navigation and flexible avoidance of local optima, thereby improving the method’s global optimization within path planning and increasing the likelihood of identifying the global optimal path.

(3) Enhanced Robotic Adaptability. The inclusion of distance factors, virtual target points, velocity factors, and acceleration factors enables the enhanced artificial potential field method to adapt more flexibly across diverse environments and tasks. This heightened adaptability facilitates superior accommodation of complex scenarios and dynamic environments, ultimately enhancing the robot’s adaptive capabilities.

(4) Reduced Path Length and Time. Through refined path planning and enhanced obstacle avoidance capabilities, the improved artificial potential field method effectively generates shorter and more efficient paths. Consequently, operational time and energy consumption are minimized, prolonging operational duration.

## 6. Experiment Verification

**Figure 19:**
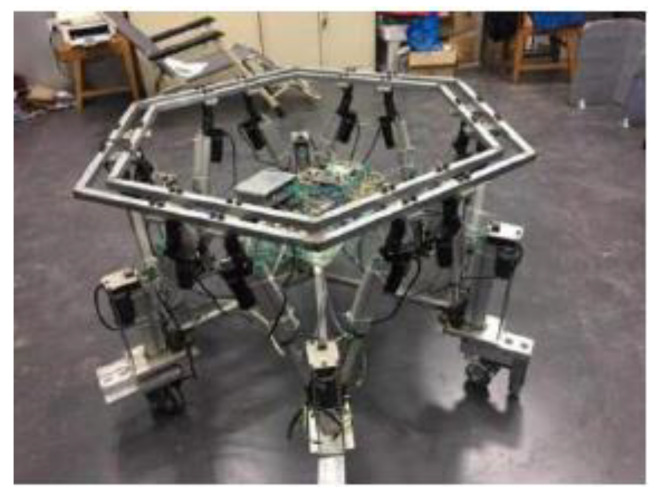
Wheel foot hybrid parallel leg walking robot.

To validate the correctness of the proposed IAPF path planning method applied to the motion simulation results of the wheel-foot hybrid parallel-leg walking robot, an experimental prototype was constructed, as shown in Figure 19. The Stm32f103 serves as the core processor, and wireless transceiver modules enable communication with the host computer. The effectiveness of the IAPF method in real-time path planning for the robot was verified by designing an obstacle-filled environment.

### 6.1. Hardware System Construction

The robot path planning algorithm is executed on a computer, serving as the upper computer control platform for the robot. The computer processes information from laser radar sensors, ultra-wideband (UWB) positioning devices, and control commands, converting this information into position and posture changes for the robot. The control signal for the wheel-foot hybrid parallel-leg walking robot is then calculated through kinematics and sent to the STM32 main control board, enabling real-time motion control of the robot.

The UWB positioning device uses four base stations as the reference coordinate system for positioning. It regards the center of these four base stations as the coordinate system’s origin and takes the tag as a fixed or moving point within this coordinate system. This device can provide real-time position information relative to the coordinate center. To facilitate the measurement of the robot’s actual position during experiments, three positioning base stations are fixed at the corners of the outdoor experimental obstacle environment, creating a virtual coordinate environment. Additionally, a label is affixed to the center of the robot platform to continuously measure the robot’s position coordinates within the obstacle environment. This information is recorded to assist in real-time algorithm calculations and viewing of experimental results.

### 6.2. Establishment of Experimental Environment

**Figure 20:**
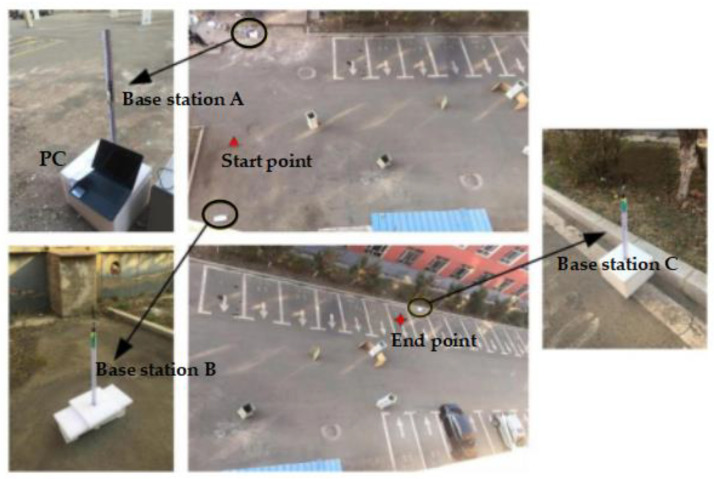
Obstacles environment and positioning base station.

The robot’s upper platform measures approximately 1 m × 1 m. As a result, the experimental obstacle environment is configured as a rectangular area measuring 15.5 m × 25 m, as illustrated in Figure 20.

**Figure 21:**
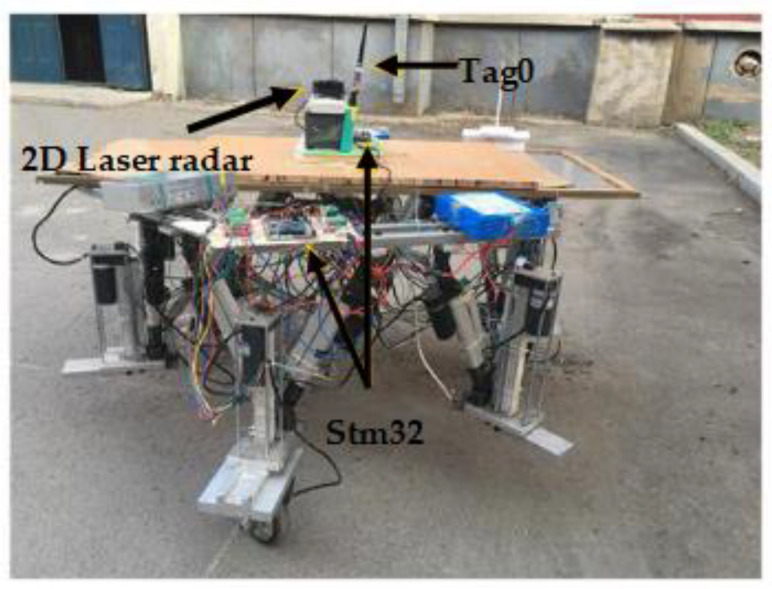
Robot in the experiment.

Three UWB positioning base stations are strategically placed at three corners of the obstacle area. Base station A serves as the primary station, and is connected to a PC for real-time communication with the robot controller. Base stations B and C assist base station A in creating a two-dimensional positioning environment. In this environment, base station A serves as the origin, AB forms the *X*-axis, and AC forms the *Y*-axis. The coordinate system’s starting point is established as (10 m, 0 m), while the ending point is set at (5 m, 21 m). The obstacle environment includes cars and randomly placed commodities. Tag0 is used for real-time robot positioning and is fixed at the center of the robot’s upper platform, as depicted in Figure 21.

### 6.3. Experiment Verification Based on IAPF Algorithm

**Figure 22:**
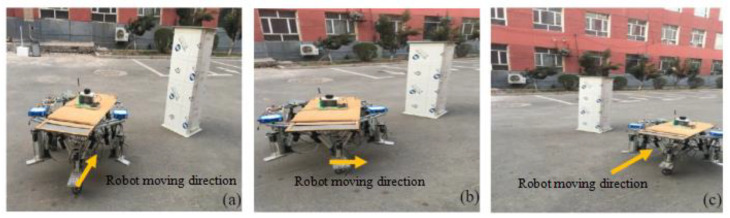
Robot avoids static obstacles. (**a**) Encounters the static obstacle. (**b**) Changes the moving direcction. (**c**) Avoids the static obstacle.

The robot commences from the starting point at coordinates (10 m, 0 m), moving at a speed of 0.5 m/s. The algorithm’s sampling time is set to 0.1 s, with an obstacle influence radius of 2 m and a minimum safe collision distance of 1 m. As shown in Figure 22, when confronted with a static obstacle, the robot effectively changes its path to avoid the obstacle.

**Figure 23:**
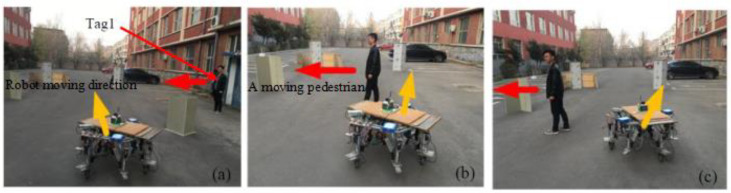
Robot avoids the moving pedestrians. (**a**) Moves and avoids the obstacles. (**b**) Encounters a moving pedestrian. (**c**) Avoids moving pedestrian.

In order to further verify the dynamic performance of the IAPF algorithm used, Tag1 is placed on a pedestrian with a speed of 0.3 m/s, which moves back and forth along a fixed path to simulate the impact of dynamic obstacles on the robot. As shown in Figure 23, when facing a pedestrian obstacle coming from the side, the robot made a turn around in due time and successfully avoided the moving pedestrians.

**Figure 24:**
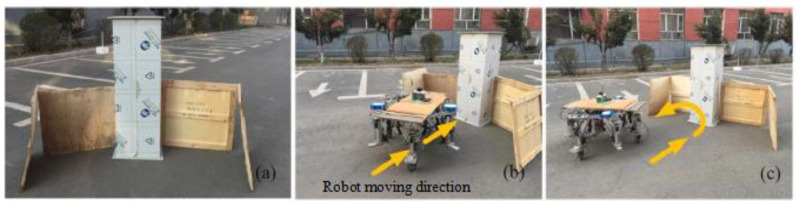
Robot escapes U-shaped trap.(**a**) U-shaped obstacles. (**b**) Sinks into U-shaped obstacles. (**c**) Escapes from the U-shaped obstacle area.

To validate the efficacy of the IAPF algorithm in addressing obstacles characterized by a U-shaped obstacle configuration, a U-shaped obstacle zone is instituted. This zone is circumscribed by both vertical plates and columns, as depicted in Figure 24a. The designated target point lies directly behind this U-shaped obstacle region. As illustrated in Figure 24b, in the absence of a preprocessing obstacle environment mechanism, the robot becomes ensnared within the confines of the U-shaped obstacle zone. Conversely, as shown in Figure 24c, by virtue of path planning employing the IAPF algorithm, the robot adeptly negotiates the U-shaped obstacle terrain and successfully extricates itself from the U-shaped snare.

### 6.4. Analysis of Experiment Results

**Figure 25:**
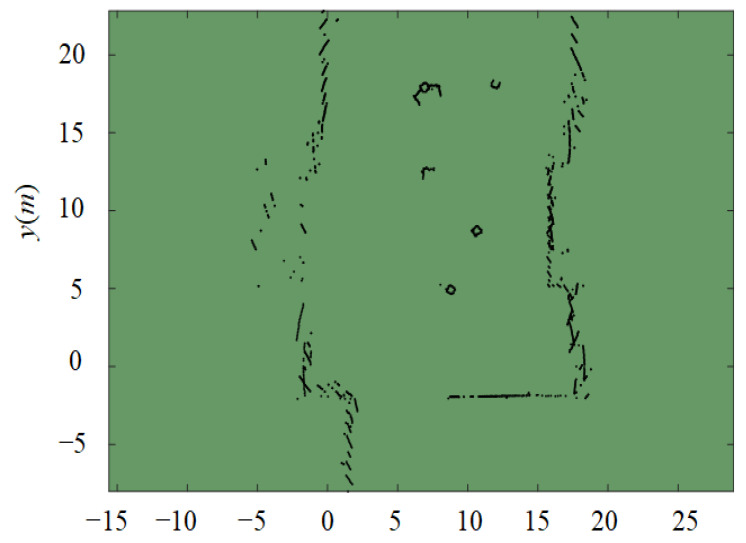
Obstacle environment acquired by 2D laser radar.

**Figure 26:**
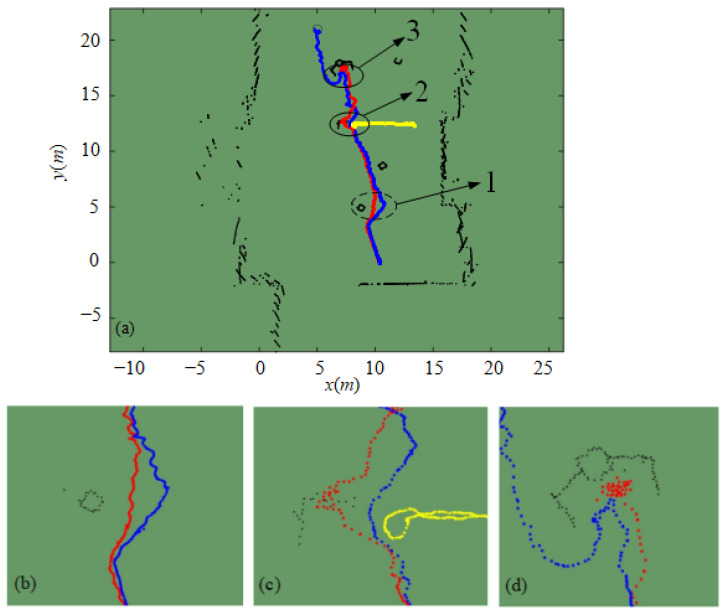
Obstacle avoidance in experiment. (**a**) Obstacles environment with robot acquired by 2D laser radar. (**b**) Static obstacle avoidance experiment. (**c**) Dynamic obstacle avoidance experiment. (**d**) U-Shaped obstacle area escape experiment.

Real-time data pertaining to obstacles is collected via a two-dimensional (2D) laser radar bearing the Delta-1A nomenclature. This data is subsequently employed to generate a 2D discrete dotted obstacle environment image. The compiled obstacle coordinates are then input into Matlab to produce an illustration of the obstacle environment, as presented in Figure 25. Throughout the course of the experiment, the UWB positioning devices are enlisted to gather real-time positional data for Tag 0 and Tag 1. This data is then wirelessly transmitted to the upper PC. When compared with the obstacle data accrued via the 2D laser radar, the experimental outcomes for both the classical APF algorithm and the IAPF algorithm can be derived, as portrayed in Figure 26.

Figure 26a visually represents the intended trajectory traced by the IAPF algorithm expounded in this paper, which is denoted by the blue path. The red trajectory signifies the planned course executed by the classical APF algorithm. The yellow path traces the trajectory of dynamic obstacles emulating pedestrians. Figure 26b–d correspond to numerical indices 1, 2, and 3 in Figure 26a. In the context of extensive spatial separation from the target location, as evinced in Figure 26b, the IAPF algorithm is obviously better than APF in collision avoidance. Although the path planned by APF algorithm can avoid dynamic obstacles confronted by the robot (see Figure 26c), it collides with static obstacles, and the trajectory planned by the IAPF algorithm makes the robot successfully avoid dynamic and static obstacles. Finally, in the face of U-shaped obstacles, as elucidated in Figure 26d, the APF algorithm causes the robot to become trapped and unable to escape; in contrast, the path planned by the IAPF algorithm enables the robot to successfully escape from the U-shaped area and reach the destination. 

The empirical findings conclusively validate the prototype’s ability to effectively circumvent static obstacles, dynamic obstacles, and U-shaped obstacles. These results, in conjunction with the simulation outcomes, further underscore the soundness and viability of the IAPF methodology in the context of collision avoidance, dynamic obstacle evasion, and extrication from U-shaped obstacle regions.

**Figure 27:**
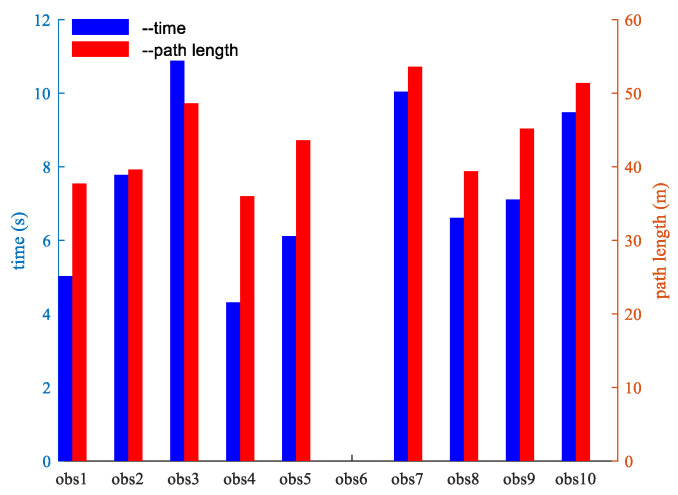
Running time and path length of robots in different obstacle environments.

To further scrutinize the algorithm’s robustness and stability, under the stipulation of uniform algorithm parameters and consistent starting and ending points within the experimental arena, we conducted experiments involving 10 distinct obstacle environments. These environments were fashioned by varying factors such as the quantity, dimensions, and arrangement of static obstacles, as well as the speed and direction of dynamic obstacles. We collected data on the robot’s operational time and path length across these 10 scenarios, as illustrated in Figure 27. The figure distinctly indicates that different configurations of obstacle environments do exert a discernible influence on the algorithm’s computation time and path length outcomes. The 90% success rate in path planning, to a considerable extent, attests to the enhanced algorithm’s robustness.

This research endeavors to enhance the parameters governing gravitational and repulsive forces within the framework of the classical APF algorithm. Nonetheless, it is imperative to acknowledge that the IAPF algorithm, by virtue of its intrinsic nature as a local path planning algorithm, inherits certain limitations similar to those encountered in conventional artificial potential field methods. Particularly when faced with intricate environmental contexts, instances of path planning failures may ensue.

## 7. Conclusions and Future Works

The present study introduces the design of a wheel-foot hybrid parallel-leg walking robot. In response to the inherent challenges associated with the classic APF methodology, an array of ameliorative approaches are posited for the robot’s path planning. The IAPF methodology is exhaustively simulated and scrutinized, affording a basis for comparative analysis vis-à-vis the classical APF methodology. The simulation outcomes substantiate the capacity of the IAPF methodology to mitigate issues concerning collision avoidance, unreachable targets, local minima, and dynamic planning insufficiencies. In tandem with these computational simulations, empirical experiments are conducted, wherein measured data is fitted to theoretical predictions, further underscoring the soundness and feasibility of the proposed theoretical model.

However, it is important to recognize that the IAPF algorithm may exhibit limited planning capabilities when confronted with more intricate obstacle scenarios. Consequently, future research endeavors may explore the combination of intelligent algorithms, such as ant colony algorithms, particle swarm algorithms, A* algorithms, and the like, to engender a hybrid algorithmic framework. Moreover, efforts may be directed toward enhancing the robot’s real-time path planning capabilities in complex environments by harnessing SLAM-based terrain construction technology.

## Figures and Tables

**Table 1:** Main parameter settings.

Main Parameters	Value
Influence area of obstacle *ρ*_0_ (m)	2
Coefficient *k_ax_*, *k_av_*, *k_aa_*	200
Coefficient *k_rx_*, *k_rv_*, *k_ra_*	1000
Distance threshold *d*_0_ (m)	2
positive exponent *n*	2
Robot detection range *R*_0_ (m)	2.5
Minimum safe collision distance *S* (m)	1
Robot body radius *r* (m)	0.5
Robot velocity *V_rob_* (m/s)	0.2
Dynamic goal velocity *V_gd_* (m/s)	0.1
Dynamic obstacle velocity *V_od_* (m/s)	0.1
*Δf*	0.02
*ΔP*	0.01

**Table 2:** Comparison of simulation results of two algorithms.

	Pursuing Dynamic Goal and Avoiding Dynamic Obstacles	
	Classic APF Method	IAPF Method
time (s)	3.49	2.47
path length (m)	22.40	15.40
path length reduction rate (%)	31.25	
Run time reduction rate (%)	29.22	

## Data Availability

No new data were created or analyzed in this study. Data sharing is not applicable to this article.
